# Protocol for an economic evaluation and budget impact assessment of a randomised, stepped-wedge controlled trial for practice change support to increase routine provision of antenatal care for maternal alcohol consumption

**DOI:** 10.1186/s43058-020-00079-5

**Published:** 2020-10-15

**Authors:** Penny Reeves, Zoe Szewczyk, Melanie Kingsland, Emma Doherty, Elizabeth Elliott, Adrian Dunlop, Andrew Searles, John Wiggers

**Affiliations:** 1 Hunter Medical Research Institute (HMRI), Lot 1, Kookaburra Circuit, New Lambton Heights, New South Wales 2305 Australia; 2 School of Medicine and Public Health, The University of Newcastle, University Drive, Callaghan, New South Wales 2308 Australia; 3 Hunter New England Population Health, Hunter New England Local Health District, Locked Bag 10, Wallsend, New South Wales 2287 Australia; 4 Faculty of Medicine and Health and Discipline of Child and Adolescent Health, University of Sydney, Sydney, New South Wales Australia; 5 Sydney Children’s Hospital Network, Kids’ Research Institute, Westmead, New South Wales Australia; 6 Drug and Alcohol Clinical Services, Hunter New England Local Health District, Newcastle, New South Wales Australia

**Keywords:** Cost-effectiveness analysis, Health economics, Antenatal care, Protocol, Budget impact assessment

## Abstract

**Background:**

Antenatal clinical practice guidelines recommend routine assessment of women’s alcohol consumption during pregnancy. The delivery of advice and referral when necessary are also recommended. However, evidence suggests there are barriers to the uptake of best-care guidelines. Effective, cost-effective and affordable implementation strategies are needed to ensure the intended benefits of guidelines are realised through addressing identified barriers. This paper describes the protocol for evaluating the efficiency and affordability of a practice change intervention compared to the usual practice in an implementation trial.

**Methods:**

The effectiveness of the intervention will be evaluated in a stepped-wedge randomised controlled implementation trial, conducted in an Australian setting. An economic evaluation will be conducted alongside the trial to assess intervention efficiency. A budget impact assessment will be conducted to assess affordability. The prospective trial-based economic evaluation will identify, measure and value key resource and outcome impacts arising from the multi-strategy practice change intervention compared with usual practice. The evaluation will comprise (i) cost-consequence analyses, where a scorecard approach will be used to show the costs and benefits given the multiple primary outcomes included in the trial, and (ii) cost-effectiveness analyses, where the primary outcome will be incremental cost per percent increase in participants reporting receipt of antenatal care for maternal alcohol consumption consistent with the guideline recommendations. Intervention affordability will be evaluated using budget impact assessment and will estimate the financial implications of adoption and diffusion of this implementation strategy from the perspective of relevant fundholders. Results will be extrapolated to estimate the cost and cost-effectiveness of rolling out the model of care.

**Discussion:**

Uptake of clinical guidelines requires practice change support. It is hypothesized that the implementation strategy, if found to be effective, will also be cost-effective, affordable and scalable. This protocol describes the economic evaluation that will address these hypotheses.

**Trial registration:**

Australian and New Zealand Clinical Trials Registry, ACTRN12617000882325. Registered on 16 June 2017

Contributions to the literature

- Research has shown the development and dissemination of clinical guidelines alone are insufficient to change practice and deliver improved patient outcomes. Further investment in strategies to increase adoption of guideline recommendations is required.
- Given escalating healthcare costs and constrained budgets in public health systems, effective, cost-effective and affordable implementation strategies are needed to ensure the intended benefits of clinical guidelines are realised.
- This protocol details the research methods that will be used to answer the following question: From the Australian healthcare system perspective, what is the cost and cost-effectiveness of the practice change intervention to increase the routine provision of antenatal care for maternal alcohol consumption compared to usual practice, and is it an affordable model for local health services?

## Background

To prevent the potential adverse obstetric and foetal outcomes associated with women’s alcohol consumption during pregnancy [1], clinical practice guidelines recommend that clinicians routinely assess alcohol consumption and advise all pregnant women that it is safest not to consume alcohol during pregnancy and of the potential harms associated with consumption. Guidelines also recommend clinicians refer to specific services when required [2]. It is also recommended that follow-up care is provided during subsequent antenatal visits. Despite these clear recommendations, the provision of routine antenatal care addressing maternal alcohol consumption during pregnancy is limited [3]. For example, in Canada, approximately 50% of health professionals have reported providing advice to pregnant women regarding the consumption of alcohol [4], and in the UK, two thirds of women reported receiving such advice from a midwife [5]. A 2005 Australian study of 1143 health professionals who provide antenatal care found that fewer than half (45%) routinely asked about alcohol consumption during pregnancy, 25% provided information on the effects of alcohol consumption during pregnancy and only 13% provided advice consistent with national drinking guidelines that recommended no alcohol consumption during pregnancy [6, 7]. A more recent study involving 166 midwives in Western Australia found that while almost all midwives (93%) asked pregnant women about their alcohol consumption, just over half (54%) used a recommended standardised assessment tool to do so (AUDIT or AUDIT-C) [7–9]. In a recent survey of women who had recently visited public antenatal services in the Hunter New England local health district, Australia, less than two thirds (64%) of pregnant women reported that they received an assessment of their alcohol consumption and just over one third (35%) received advice and referral appropriate to their self-reported level of alcohol consumption since pregnancy recognition at their initial antenatal visit [9]. Less than 10% of women received such care at subsequent antenatal visits [9].

The development and dissemination of clinical guidelines alone are insufficient to change the current practice and deliver improved patient outcomes [10]. Further investment in strategies to increase adoption of guideline recommendations is required [11]. However, decisions about implementation intervention investment should be guided by consideration of effectiveness as well as economic efficiency, equity and affordability [12, 13]. Economic evaluation combines evidence about the cost and benefits of alternative interventions in order to identify investment opportunities that demonstrate value for money [14–16]. Given escalating healthcare costs and constrained budgets in public health systems, economic evaluations contribute significantly to the evidence base informing decision-makers and healthcare funders. Effective, cost-effective and affordable implementation strategies are needed to ensure the intended benefits of clinical guidelines are realised [17]. Similarly, assessment of the budget impact of implementation strategies is warranted to assess the affordability and financial consequences of healthcare practice changes. At present, there is limited evidence regarding the economic cost of adverse foetal and maternal outcomes associated with alcohol consumption during pregnancy [18–21] and no evidence concerning the cost-effectiveness of practice change interventions aiming to improve recommended antenatal care for maternal alcohol consumption [22]. This paper presents a protocol for the economic evaluation of an antenatal practice change intervention to improve care addressing alcohol consumption in pregnancy. The paper aims to answer the following research question: From the Australian healthcare system perspective, what is the cost and cost-effectiveness of the practice change intervention to increase the routine provision of antenatal care for maternal alcohol consumption compared to usual practice, and is it an affordable model for local health services?

## The trial

### Study design

The multi-strategy practice change implementation trial will be a randomised, stepped-wedge controlled trial. The protocol has been previously published [3]. In brief, the trial will be conducted in all public antenatal services within three sectors across two health districts in New South Wales, Australia. The model of care for addressing alcohol consumption by pregnant women will be delivered to sectors in a random, stepped order. The main outcomes are described below in the “Identification and measurement of outcomes” section and described in detail in Kingsland et al. [3].

Repeated cross-sectional outcome data will be gathered on a weekly basis across the three sectors for a period of 34 months. Baseline data collection, representing usual practice (control), will be collected for the three sectors from 7 months prior to the commencement of the intervention in the first sector to the start of the intervention in each sector. Follow-up data will be collected for the three sectors 7 months following completion of the intervention in the third and last sectors. The outcome results will be determined by comparing practice change outcomes between the baseline and follow-up periods for the three sectors combined.

### Usual practice

Usual practice comprises the antenatal care for addressing maternal alcohol consumption during pregnancy that is provided in the baseline period prior to the introduction of the intervention. It is anticipated that such care is likely to vary by antenatal service and clinician. This is due to variability in local practice across the 3 sectors covering metropolitan, regional and rural localities, as well as the lack of an existing health district-wide guideline or procedure specifying the provision of routine care for addressing alcohol consumption during pregnancy.

The development and dissemination of the clinical practice guidelines have already taken place in Australia with the result that their associated costs and effects are common to both intervention and control study periods.

### The intervention

A multi-strategy practice change intervention has been developed to support antenatal care staff to implement a model of care consistent with clinical guidelines. The multiple strategies included in the intervention are presented in Appendix 2: Table 1. The model of care is based on an evidence-informed behavioural counselling framework [23] and includes clinician assessment of patient alcohol risk status using the AUDIT-C tool at the first comprehensive (‘booking in’) visit and at follow-up antenatal appointments at 27–28 weeks and 35–36 weeks gestation. All pregnant women will be provided with brief advice that it is safest not to consume alcohol during their pregnancy and of the risks associated with alcohol consumption at this time. Women who are at ‘medium risk’ of harm according to their AUDIT-C score of 3–4 will be offered a referral to the New South Wales Get Healthy in Pregnancy Service, an evidence-based telephone coaching service provided free of charge. Women at ‘high risk’ of harm from alcohol (AUDIT C score, 5+) will be referred to the Hunter New England Drug and Alcohol Clinical Service.

## Methods and analysis

This economic evaluation has been conducted and reported in accordance with the Consolidated Health Economic Evaluation Reporting Standards (CHEERS) publication guidelines and good reporting practices [24].

### Economic evaluation overview

Cost, cost-consequence and cost-effectiveness analyses will be undertaken comparing the intervention against the usual practice from a public health service perspective. This perspective is justified because ongoing funding for this intervention, especially if it translates into routine practice, will fall on public health services. To further aid decision-makers, budget impact analysis, including scale-up cost scenarios, will be presented alongside the cost-effectiveness findings. Costs will be reported in 2019 Australian dollars ($AUD). The time horizon for the inclusion of relevant costs and consequences will be the course of the trial (34 months). Costs and benefits occurring after 12 months will be discounted using an annual discount rate of 3% in the base case. Annual discount rates of 0 and 5% will be applied in a sensitivity analysis. The conduct, analysis and reporting of the economic analyses will adhere to the cost and economic analysis guidelines [14, 15, 25] and Consolidated Health Economic Evaluation Reporting Standards guidelines [15].

Common to all forms of economic evaluation is the analysis of cost. In this study, costing and budget impact assessments will be conducted to quantify how much more it will cost to pursue implementation efforts to affect practice change. The budget impact assessment will translate the health economic findings into more meaningful and relevant results for healthcare decision-makers and funders. In its simplest form, economic evaluation involves a listing of all cost/benefit implications of the alternatives under consideration, as in cost-consequences analyses [26]. A cost-consequence analysis is employed in this analysis because it provides information for spending decisions when implementation strategies are complex and are expected to have outcomes that are too disparate to be combined meaningfully. In this trial, there are four primary outcomes (see the “Identification and measurement of outcomes” section). Cost-consequence analyses permit value judgements without having to fully specify a relation between all the different measures of outcomes [11]. Cost-effectiveness will depend on the effect of the intervention on care provider behaviour. The greater the difference in expected outcomes between usual practice and the new model of care, and the more widespread the implementation, the more likely a strategy is to be cost-effective. In this study, the likelihood of achieving an outcome difference will be maximised by using a staged process to both understand the barriers to guideline adoption and to develop the implementation strategies [3]. All public antenatal services in the three sectors will receive the practice change intervention, including midwifery group practices, midwifery clinics, specialist medical services, Aboriginal Maternal and Infant Health Services (AMIHS) and multi-disciplinary teams caring for women with complex pregnancies or identified vulnerabilities.

### Trial-based economic evaluation and budget impact assessment

#### Identification and measurement of outcomes

It has been suggested that one of the ways to improve efficiency in conducting economic evaluations of implementation interventions is to confine studies to measures of the care process or intermediate outcomes [11], for example, change in professional guidance adherence or compliance [3]. This approach is based on the premise that the guideline recommendations are cost-effective in and of themselves. In this study, the outcome measures are confined to the care process for efficiency. The implementation trial has four primary outcomes. They are the proportion of all antenatal clinic appointments (at ‘booking in’, 27–28 weeks gestation and 35–36 weeks gestation) for which women report the following:

1. Being assessed for alcohol consumption and level of risk using the AUDIT-C
2. Being provided with brief advice related to alcohol consumption during pregnancy
3. Receiving, relative to their level of risk, the relevant elements of antenatal care for addressing alcohol consumption during pregnancy (advise and refer)
4. Being assessed for alcohol consumption and level of risk using the AUDIT-C and receiving, relative to their level of risk, the relevant elements of antenatal care for addressing alcohol consumption during pregnancy (advise and refer)

Receipt of care will be measured by participant report during a computer-assisted telephone survey conducted after an antenatal consultation, at each of the three time points [3].

A secondary outcome will also be included. For women attending antenatal appointments at ‘booking in’, 27–28 weeks gestation and 35–36 weeks gestation, alcohol consumption since pregnancy recognition will be collected. Outcome measurement will be based on self-report of women using the total AUDIT-C score. AUDIT-C is a validated tool for assessing the risk of harm due to alcohol consumption [27].

#### Identification, measurement and valuation of costs

Cost data pertaining to the development and implementation of the practice change intervention will be collected prospectively using a resource use capture tool in tangent with trial administrative records. The intervention programme logic will be used to identify all the relevant costs directly and indirectly associated with the intervention. The cost capture tool, developed in Microsoft Excel (2013), allows researchers to prospectively document the activity and materials consumed at different phases of the intervention (development, immediate execution and maintenance) from all relevant stakeholders. The cost capture tool includes the following categories: (1) labour (health service and non-health service staff, including overheads to allow for additional costs of employment), (2) materials (non-labour cost items such as stationary, education materials, electronic hardware or software), (3) joint costs (incurred in connection with multiple projects, for example, the maintenance costs of a website portal supporting different interventions; capital costs such as one-off investments such as the purchase of additional office buildings or motor vehicles), and (4) miscellaneous costs (which include costs not easily classified into the other categories, for example, venue hire, travel and overnight accommodation). To maintain a conservative approach to cost estimation, the non-capital implementation costs will not be amortised.

Resource use valuation will be based on the concept of opportunity cost, that is, the value of the benefit forgone in not employing a resource for a different use. Market prices will be used as a proxy for the ‘value of benefit’ forgone [28].

#### Costing study

Appendix 3: Table 2 summarises the costs expected to be included in the study. The cost analysis will use measures of arithmetic means, between-group differences and variability of differences [29, 30]. Costs will be calculated individually for each sector in the trial, as well as aggregated across all sites. Intervention component costs will be disaggregated to provide insight into the cost of individual practice change intervention strategies.

#### Cost-consequence, cost-effectiveness and equity

**Fig. 1:**
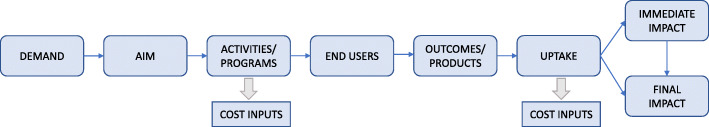
Example intervention programme logic

As outlined above, the range of outcomes measured in the implementation trial is diverse, which lends well to a cost-consequence analysis. The analysis will adopt a scorecard approach to show a comparison of the costs and benefits associated with the intervention and usual practice. An economic summary measure is not calculated. A programme logic model will be developed to describe all possible inputs (costs) and impacts (consequences) associated with the intervention and usual care (Appendix 1: Fig. 1).

The cost-effectiveness analysis will be conducted subject to the assessment of intervention efficacy. The economic summary measure will be an incremental cost-effectiveness ratio (ICER). The ICER represents the additional cost required to achieve an additional unit of benefit [14, 29]. For this study, the ICER will be calculated as the incremental cost per percent change in the proportion of participants reporting that receipt of ‘antenatal care for maternal alcohol consumption consistent with guideline recommendations’ was provided to them during their antenatal consultation.

Distributional cost-effectiveness analysis (DCEA) is a framework for incorporating health inequality concerns into the economic evaluation of health sector interventions. Full DCEA requires the distribution of direct health benefits to be estimated from a decision-analytic model or trial-based analysis using parameter estimates specific to socioeconomic groups. However, a simplified version providing healthcare decision-makers and stakeholders with an evidence-based technique for evaluating whether new interventions can help to achieve the objective of health inequality reduction can be used when conducting a full DCEA is not practical or feasible [31]. To assess the equity implications of the intervention, the use of distributional cost-effectiveness will be explored in the scenario examining scale-up subject to the availability of requisite data [31, 32].

#### Budget impact assessment

Economic evaluations and budget impact analyses share many of the same data elements and methodological requirements and should be viewed as complementary. However, there are important differences in their methods and use cases [13]. The budget impact assessment will translate the results of the economic costing study into financial consequences relevant to decision-makers and fundholders within the health districts.

A model will be developed to describe the financial resources associated with the usual practice over the course of health districts’ budgeting cycles. This will represent the base case or ‘reference case’. The comparative scenario will model the required changes in health service resourcing that are expected to result from the adoption of this alternate model of care, including indirect and downstream impacts on other parts of the health service. Resource use data will be sourced from the implementation trial and costing analysis. All model assumptions and data inputs will be described in full. Justification for the inclusion or exclusion of relevant model parameters will be provided.

The budget impact assessment will adhere to the relevant local and international guidelines, as well as recommended formats for presenting the results so they are most useful to decision-makers [13, 33].

### Sensitivity and uncertainty analyses

All analyses will be subject to one-way and probabilistic sensitivity analyses. These analyses test the impacts of plausible variation in data parameters on the cost outcomes and economic summary measure and provide an understanding of which values are associated with the greatest amount of uncertainty. Differences in costs or outcomes that can be explained by variations between subgroups of patients with different baseline characteristics or other observed variabilities in effects that are not reducible by more information will be reported.

In addition, scenario analyses will be undertaken to explore the efficiency and budget impact of the state-wide implementation of the practice change model of care in maternity services across the whole state of NSW.

## Discussion

This protocol sets out the plan to assess the cost, efficiency and affordability of a multi-strategy practice change implementation intervention compared to usual practice. The purpose of publishing this protocol is twofold; first, setting an a priori plan for the proposed analyses can reduce potential biases made from ad hoc analytic decisions. Deviations from this protocol will require description and justification in the final analyses. Second, there are benefits to the research and broader community in a greater understanding of economic evaluation, especially with respect to their conduct alongside implementation trials. There is a clear absence of research evidence of the effectiveness, cost, cost-effectiveness and budget impact of implementation strategies to improve antenatal care that addresses maternal alcohol consumption during pregnancy [34]. The application of economic evaluation to health-promoting, implementation interventions is limited [12] while the application of budget impact assessment at the local health service level is completely novel. This will be the first economic evaluation and budget impact assessment of an implementation strategy in this field [22]. It is expected that the practice change intervention will increase the extent to which women are assessed for alcohol consumption during pregnancy and given evidence-based advice and, where appropriate, referral to ongoing support services to avoid the consumption of alcohol for the remainder of their pregnancy. The outcomes of these analyses will then inform the state-wide scale-up of this implementation intervention and the next step in the research translation pathway.

## Conclusion

This protocol outlines the assessment of cost, efficiency and affordability of a multi-strategy practice change implementation intervention compared to the usual practice. The outcomes of this economic evaluation will provide insight into the cost, cost-consequence and cost-effectiveness of implementation strategies designed to improve antenatal care addressing the recognised risk of alcohol consumption to the health and wellbeing of both the mother and child [34], and inform future healthcare policy, investment allocation and research.

## Data Availability

There are no datasets associated with this protocol.

## Appendix 2

**Table 1:** Implementation strategy summary

Intervention component	Component details: *A full description of component details has been published elsewhere* [3].
**Leadership and management**	• Monthly meetings will be held with management from antenatal services to elicit support. • Service managers will be asked to distribute resources to staff and attend training sessions. • Monitoring and reporting of performance measures related to the intervention.
**Local clinical practice guidelines**	• A service-level guideline and procedure document will detail the model of care, including assessment, brief advice and referral pathways. • This document will be uploaded onto the health service’s policy directory, disseminated by managers to all staff via email, and hard copies will be placed in staff common areas.
**Electronic prompt and reminder system**	• Existing point-of-care and medical record systems used by maternity clinicians will be modified to electronically prompt the use of the AUDIT-C alcohol screening tool. • Brief advice scripts will be displayed on the point-of-care system based on the woman’s AUDIT-C risk score, and prompts and tools for referral to appropriate services.
**Local opinion leaders/champions**	• Project-specific clinical midwife educators appointed to support staff to uptake the model of care and provide support at a one-on-one, team and service level. • Additional local antenatal clinical leaders will be engaged to provide encouragement and demonstrate required behaviours as required.
**Educational meetings and materials**	• Training will be provided to all antenatal service clinicians via a 30-min online training module and face-to-face sessions. Clinical midwife educators will facilitate clinicians in completing the online training and coordinate face-to-face training sessions. This will include lecture-style sessions, interactive sessions, case study-based sessions and one-on-one sessions. • Clinicians will be provided with written resources (hardcopy and electronic) to support the model of care, including standard drink measure charts and point-of care written prompts/reminders (e.g. stickers in charts).
**Academic detailing**	• Data from both medical records and telephone surveys conducted with women who attended the antenatal services will be used to provide feedback on adherence to the agreed model of care. • The clinical midwife educators will visit service teams in their antenatal clinics to provide feedback data and develop action plans to improve adherence.
**Monitoring and accountability**	• Antenatal service managers will report, interpret and monitor performance measures for the model of care. • These results will be disseminated to antenatal service staff through team meetings, emails and other usual communication mechanisms. • Performance measures will be built into the existing monitoring and accountability frameworks for antenatal services.

## Appendix 3

**Table 2:** Description of resource use data for inclusion in the economic evaluation

Intervention component	Resource use details	Data collection method
**Intervention strategy development**	• Labour time: health district project/implementation support officer time. • Materials.	• Resource use capture template
**Leadership and management**	• Labour time: health district project/implementation support officer time, health service clinical staff (management from antenatal services).	• Resource use capture template
**Local clinical practice guidelines**	• Materials: guideline and procedure document development and provision. • Electronic dissemination.	• Resource use capture template
**Electronic prompt and reminder system**	• Materials: online/computer-based intervention component. • Electronic dissemination.	• Resource use capture template
**Local opinion leaders/champions**	• Labour time: change champion, clinicians and trainers.	• Resource use capture template
**Educational meetings and materials**	• Labour time: health district project/implementation support officer time, health service clinical staff. • Materials: educational tools and resources.	• Resource use capture template
**Academic detailing**	• Labour time: project support officer, clinical service staff time.	• Project administrative records • Resource use capture template • REDCap self-report survey
**Monitoring and accountability**	• Antenatal service managers will report, interpret and monitor performance measures for the model of care. • These results will be disseminated to antenatal service staff through team meetings, emails and other usual communication mechanisms. • Performance measures will be built into the existing monitoring and accountability frameworks for antenatal services.	• Resource use capture template

## Abbreviations

AUD: Australian dollars
AUDIT-C: Alcohol Use Disorders Identification Test
BIA: Budget impact assessment
CEA: Cost-effectiveness evaluation
DCEA: Distributional cost-effectiveness analysis
ICER: Incremental cost-effectiveness ratio

## References

[1] Bower C, Elliott E. on behalf of the Steering Group. Report to the Australian Government Department of Health: “Australian guide to the diagnosis of fetal alcohol spectrum disorder (FASD)”. 2016.

[2] World Health Organization (WHO). Guidelines for the identification and management of substance use and substance use disorders in pregnancy. 2014, WHO: Geneva. 24783312

[3] Kingsland M, Doherty E, Anderson AE, Crooks K, Tully B, Tremain D, et al. A practice change intervention to improve antenatal care addressing alcohol consumption by women during pregnancy: research protocol for a randomised stepped-wedge cluster trial. Implement Sci. 2018;13(1):N.PAG-N.PAG. 10.1186/s13012-018-0806-x PMC6102816 30126437

[4] Canada H (2003). A national survey regarding knowledge and attitudes of health professionals about fetal alcohol syndrome.

[5] Health and Social Care Information Centre. Infant feeding survey. Health and Social Care Information Centre; 2007.

[6] Payne JM, Elliott E, D’Antoine HA (2005). Health professionals’ knowledge, practice and opinions about fetal alcohol syndrome and alcohol consumption in pregnancy. Aust NZ J Public Health..

[7] Elliott E, Payne JM, Haan E (2006). Diagnosis of fetal alcohol syndrome and alcohol use in pregnancy: a survey of paediatricians’ knowledge, attitudes and practice. J Paediatr Child Health..

[8] Payne J, Elliott E, D’Antoine H, O’Leary C, Mahony A, Haan E (2005). Health professionals’ knowledge, practice and opinions about fetal alcohol syndrome and alcohol consumption in pregnancy. Australian and New Zealand Journal of Public Health..

[9] Doherty E, Wiggers J, Wolfenden L, Anderson AE, Crooks K, Tsang TW (2019). Antenatal care for alcohol consumption during pregnancy: pregnant women’s reported receipt of care and associated characteristics. BMC Pregnancy and Childbirth..

[10] Grimshaw JM, Eccles M, Thomas R, MacLennan G, Ramsay C, Fraser C. Toward evidence-based quality improvement. Evidence (and its limitations) of the effectiveness of guideline dissemination and implementation strategies 1966–1998. J Gen Intern Med. 2006;21. 10.1111/j.1525-1497.2006.00357.x PMC2557130 16637955

[11] Hoomans T, Severens JL. Economic evaluation of implementation strategies in health care. Implementation Science: IS. 2014;9:168. 10.1186/s13012-014-0168-y PMC4279808 25518730

[12] Reeves P, Edmunds K, Searles A, Wiggers J (2019). Economic evaluations of public health implementation-interventions: a systematic review and guideline for practice. Public Health..

[13] Mauskopf JA, Sullivan SD, Annemans L, Caro J, Mullins CD, Nuijten M, et al. Principles of good practice for budget impact analysis: report of the ISPOR Task Force on good research practices--budget impact analysis. Value in Health: the Journal of the International Society for Pharmacoeconomics and Outcomes Research. 2007;10(5):336-347. 10.1111/j.1524-4733.2007.00187.x 17888098

[14] Drummond MF, Sculpher M, Claxton K, Stoddart GL, Torrance GW. Methods for the economic evaluation of health care programmes, fourth edition. Oxford: Oxford University Press; 2015.

[15] Husereau D, Drummond M, Petrou S, Carswell C, Moher D, Greenberg D, et al. Consolidated Health Economic Evaluation Reporting Standards (CHEERS) statement. BMJ: British Medical Journal. 2013;346:f1049. 10.1136/bmj.f1049 23529982

[16] World Health Organization. Promoting health, preventing disease: the economic case. 2015.

[17] Doherty E, Kingsland M, Wiggers J, Anderson AE, Elliott EJ, Symonds I (2019). Barriers to the implementation of clinical guidelines for maternal alcohol consumption in antenatal services: a survey using the theoretical domains framework.

[18] System JFR. Food Regulation Standing Committee decision regulation impact statement: pregnancy warning labels on packaged alcoholic beverages. Canberra: Food Regulation Secretariat. 2018 October;2018.

[19] Health Technology Analysts Pty Ltd. Fetal alcohol spectrum disorder (FASD): exploratory economic analysis of different prevention strategies in Australia and New Zealand Food Standards Australia New Zealand; 2010 May, 2010.

[20] Popova S, Lange S, Burd L, Rehm J (2016). The economic burden of fetal alcohol spectrum disorder in Canada in 2013. Alcohol Alcohol..

[21] Popova S, Lange S, Burd L, Rehm J (2012). Health care burden and cost associated with fetal alcohol syndrome: based on official Canadian data. PLoS ONE..

[22] Szewczyk Z, Holliday E, Collins C, Reeves P. A systematic review of economic evaluations of antenatal nutrition and alcohol interventions and their associated implementation interventions. In: The University of Newcastle, editor. Manuscript in preparation. 10.1093/nutrit/nuaa015 32712657

[23] Whitlock EP, Green CA, Polen MR, Berg A, Klein J, Siu A, et al. U.S. Preventive Services Task Force Evidence Syntheses, formerly Systematic Evidence Reviews. Behavioral counseling interventions in primary care to reduce risky/harmful alcohol use. Rockville (MD): Agency for Healthcare Research and Quality (US); 2004. 20722127

[24] Husereau D, Drummond M, Petrou S, Carswell C, Moher D, Greenberg D, et al. Consolidated Health Economic Evaluation Reporting Standards (CHEERS)--explanation and elaboration: a report of the ISPOR Health Economic Evaluation Publication Guidelines Good Reporting Practices Task Force. Value in Health: the Journal of the International Society for Pharmacoeconomics and Outcomes Research. 2013;16(2):231-250. 10.1016/j.jval.2013.02.002 23538175

[25] National Health and Medical Research Council. How to compare the costs and benefits: evaluation of the economic evidence Canberra; 2001.

[26] McIntosh E (1999). Changing professional practice: theory and practice of clinical guidelines implementation.

[27] Burns E, Gray R, Smith LA (2010). Brief screening questionnaires to identify problem drinking during pregnancy: a systematic review. Addiction..

[28] Weinstein MC, Russell LB, Gold MR, Siegel JE (1996). Cost-effectiveness in health and medicine: Oxford University Press.

[29] Glick HD, JA, Sonnad, SS. Polsky, D. Economic evaluation in clinical trials. New York: Oxford University Press2007.

[30] Ramsey SD, Willke RJ, Glick H, Reed SD, Augustovski F, Jonsson B, et al. Cost-effectiveness analysis alongside clinical trials II-an ISPOR Good Research Practices Task Force report. Value in Health: the Journal of the International Society for Pharmacoeconomics and Outcomes Research. 2015;18(2):161-172. 10.1016/j.jval.2015.02.001 25773551

[31] Love-Koh J, Cookson R, Gutacker N, Patton T, Griffin S. Aggregate distributional cost-effectiveness analysis of health technologies. Value in Health: the Journal of the International Society for Pharmacoeconomics and Outcomes Research. 2019;22(5):518-526. 10.1016/j.jval.2019.03.006 31104729

[32] Asaria M, Griffin S, Cookson R. Distributional cost-effectiveness analysis: a tutorial. Medical Decision Making: an International Journal of the Society for Medical Decision Making. 2016;36(1):8-19. 10.1177/0272989X15583266 PMC4853814 25908564

[33] Pharmaceutical Benefits Advisory Committee. 4.5 Estimate financial implications for the health budget Australia Department of Health 2016 [Available from: https://pbac.pbs.gov.au/section-4/4-5-estimated-financial-implications-for-the-health-budget.html].

[34] Kingsland M, Doherty E, Anderson AE, Crooks K, Tully B, Tremain D, et al. A practice change intervention to improve antenatal care addressing alcohol consumption by women during pregnancy: research protocol for a randomised stepped-wedge cluster trial. Implementation Science: IS. 2018;13(1):112. 10.1186/s13012-018-0806-x PMC6102816 30126437

